# War economy and the COVID-19 pandemic: Inequalities in stimulus packages as an additional challenge for health systems

**DOI:** 10.1590/0037-8682-0245-2020

**Published:** 2020-06-01

**Authors:** Carlos Dornels Freire de Souza

**Affiliations:** 1 Universidade Federal de Alagoas, Departamento de Medicina, Núcleo de Estudos em Medicina Social e Preventiva, Arapiraca, AL, Brasil.

**Dear Editor,**

War economics refers to the set of exceptional economic measures taken during a certain period of time, generally defined by the existence of an armed conflict (war). It is characterized by the increase in public spending and centralization of economic guidelines within the scope of public power, which starts to dictate the economy’s mode of operation seeking to save it^1^.

It is worth highlighting the definition of the illustrious Dr. James Kenneth Galbraith: “*In a war economy, the public obligation is to do what is necessary: to support the military effort, to protect and defend the home territory, and especially to maintain the physical well-being, solidarity, and morale of the people. These may not prove to be easy tasks in the months ahead* ^1^.

In 2020, many countries have evoked the war economy in the face of the COVID-19 pandemic. In this case, it is a “war-free” war economy, at least in the generic sense. The war is against an invisible agent, who as of May 12, 2020 had already caused more than 280,000 deaths worldwide^2^.

The process of dissemination of COVID-19 across countries and the sanitary measures adopted to stop the expansion of the disease resulted in significant economic losses: slowdown in the global economy, rapid fall in the values of financial assets, fall in imports/exports, shrinkage of industrial production, increased inflation, reduced wages, increased unemployment and breakdown of tourism, and the provision of services^3^ ^-^ ^4^. It is evident that the damage caused is not limited to the economic sector, but alongside the health sector, they are the most affected by the pandemic.

In low- and middle-income countries, the damage is even greater, as these countries already face chronic economic and social problems. In countries like India, Brazil, and Mexico, for example, the lack of income-guarantee mechanisms for the poorest workers during the pandemic is another aggravating factor. In Brazil, the government offered timid support to workers, about R$600.00 per month for three months.

Faced with the pandemic and its consequences, many countries are turning to the term “*war economy*” to adopt measures to contain the effects of the pandemic. In absolute values: The USA envisages a US$2 trillion stimulus package; in Japan, US$1 trillion; in Germany, US$808 billion. On the other hand, developing countries such as Brazil, India, and Mexico, are offering more timid packages (< US$300 billion). Considering gross domestic product (GDP), Brazilian fiscal measures reach only 3.1% of GDP, much lower than what is observed in other countries such as the USA, Germany, Canada, and Japan^5^.

Another important component is industrial conversion^1^, which is characterized by the use of the industrial apparatus for the production of goods needed at the time of crisis, such as medical equipment. This is a strategy used by the USA in the face of the COVID-19 pandemic, with the manifest intention of forcing the automotive industry to produce ventilators to serve the population. Associated with this, many countries are banning exports on the grounds that household products are needed domestically. Given this, how can countries that lack large industry and who depend on the import of products cope? Certainly, they will have even less weapons to fight the pandemic.

It remains to be seen whether these measures will achieve their purpose or whether we will face an [almost certain] economic recession and its impact on development, especially that of low- and middle-income nations^6^. In these countries, a small stimulus package during this pandemic can create an even greater burden on the health system. This occurs for two reasons. First, the impoverishment of the population increases their vulnerability to diseases related to their squalid living conditions, such as leishmaniasis, schistosomiasis, leprosy, tuberculosis, and gastrointestinal diseases, among others^7^. There are approximately 1.2 billion people in extreme misery in the world and this poverty forces individuals to live in conditions that favor illness: lack of potable water, poor access to food, housing and the health system itself, are just some examples of this process^7^.

The second relates to the potential for countries to cope with the pandemic. With fewer resources available, it is much more difficult to guarantee comprehensive care for all individuals: adequate supply of infirmary and intensive care unit (ICU) beds, multi-professional teams properly trained and prepared to provide care to patients, health surveillance teams, supplies and medicines, and other resources necessary for comprehensive care^8^ ^-^ ^9^. 

It is necessary to highlight that health is an inalienable social right, as expressed in the Federal Constitution of Brazil (FC/1988). As such, it is the duty of the State to ensure that all citizens have access to this right through the Unified Health System (SUS, in Portuguese), based on the philosophical principles of universality, integrality, and equity^10^.

The World Health Organization recommends 16 measures to be adopted by national health systems. How they can be implemented in the face of scarce resources is still not known. They are:

1. Expand capacity for communication and proactively manage media relations.
2. Bolster capacity of essential public health services to enable emergency response.
3. Clarify first-point-of-contact strategy for possible COVID-19 cases: phone, online, physical. 4. Protect other potential first-contact health system entry points.
4. Designate hospitals to receive COVID-19 patients and prepare to mobilize surge acute and ICU capacity.
5. Organize and expand services close to home for COVID-19 response.
6. Maintain continuity of essential services while freeing up capacity for COVID-19 response. 8. Train, repurpose, and mobilize the health workforce according to priority services.
7. Protect the physical health of frontline health workers.
8. Anticipate and address the mental health needs of the health workforce.
9. Review supply chains and stocks of essential medicines and health technologies.
10. Mobilize financial support and ease logistical and operational barriers.
11. Assess and mitigate potential financial barriers to accessing care.
12. Assess and mitigate potential physical access barriers for vulnerable groups of people.
13. Optimize social protection to mitigate the impact of public health measures on household financial security.
14. Ensure clarity in roles, relationships, and coordination mechanisms in health system governance and across government^11^.

On April 7, 2020, Brazil’s Ministry of Economy launched “Brazil’s Policy Responses to COVID-19,” according to which actions to confront COVID-19 involve actions directed at the public and private sector^12^. Within the scope of the health system, 18 actions are listed. It remains to be seen whether these measures will be able to contain the spread of the pandemic and minimize its harmful consequences in the country^12^:

1. The addition of 2,000 new ICU beds and recommending that elective surgeries be postponed.
2. Health insurance firms: The National Supplementary Health Agency will list tests for COVID-19 under mandatory coverage for health insurance firms.
3. More doctors: 5,811 professionals called to join the Mais Médicos program must assume their duties. The new doctors will mostly be sent to areas of greater population concentration. Exchange medical doctors from the former Mais Médicos program will be reinstated with a deadline to start activities on 05/05/2020.
4. Telemedicine services have been allowed. Physicians can make consultations online and issue electronically signed medical reports or prescriptions.
5. Health Guarantee Fund resources: The Agency of Supplementary Health (ANS) was requested to approve measures to ease access to 20% of the Fund’s resources (about US$ 2.0 billion), providing private health insurance companies with investment funding for assistance infrastructure. Small hospitals were also authorized to treat infected patients.
6. A simplified authorization process for hygiene products was adopted by the National Agency for Health Surveillance (Anvisa) to help increase their supply.
7. Rapid test kits: The federal government and states are preparing to distribute 10 million units.
8. Temporary exemption of IPI, the Industrialized Products Tax, for listed imported and domestic goods necessary to combat COVID-19.
9. DPVAT: The Insurance for Traffic Accidents fund balance has been transferred to the public health system, an amount of R$ 4.5 billion (US$ 0.9 billion).
10. BNDES created a R$ 2 billion credit line to increase emergency capacity, medical materials, and hospital equipment.
11. Reduction of red tape on procurement procedures related to public health emergencies.
12. Government properties will be used as field hospitals, with the goal of installing structures in all capitals and large urban centers.
13. The Armed Forces’ laboratories are directed to manufacture alcohol in gel and chloroquine at an industrial scale.
14. The Ministry of Defense is registering companies that operate in the defense sector and identifying those that can provide equipment to help fight the virus.
15. Purchase of respirators (R$ 1 billion or US$ 200 million) scheduled to be delivered by the end of April.
16. R&D Financing: A fund of R$ 50 million (US$ 10 million) will be allocated to financing 11 thematic research lines, including the development of new methods of prevention and control, diagnosis, treatment, and vaccines against coronavirus and other respiratory diseases.
17. Suspension of the annual medicine price readjustment.
18. Census 2020: Postponement to 2021, transferring funds to the Ministry of Health.

Source: Brasil.^10^ Text taken entirely from Brazil’s Policy Responses to COVID-19.

Critically, the adoption of isolation and/or social distance strategies during the growth of the COVID-19 contamination curve will be beneficial to the resumption of the economy after this first wave of transmission^13^, as it can both shorten the pandemic’s duration and preserve human life, a fundamental element for the production of wealth and economic recovery post-pandemic. Interrupting social isolation at a time when contagion curves are on the rise is likely to result in an even more intense economic downturn. This is an important consensus among economists^14^.

There is no set deadline for all this to pass (whether months, years, or decades), but China offers us hope. The interruption of community transmission and decline in deaths indicates that the disease was controlled in the country, at least in this first wave of transmission. This scenario may indicate that in the second half of 2020 the global population may start to "walk out of the house*,*" after a long journey of isolation and learning. But, what about health systems? What path will they take after the pandemic? There are no easy answers, but it is a question that must be considered.
